# Serum Bone Resorption Markers after Parathyroidectomy for Renal Hyperparathyroidism: Correlation Analyses for the Cross-Linked N-telopeptide of Collagen I and Tartrate-Resistant Acid Phosphatase

**DOI:** 10.1100/2012/503945

**Published:** 2012-07-31

**Authors:** Kuo-Chin Hung, Chung-Yu Huang, Chuan-Chieh Liu, Chih-Jen Wu, Shao-Yuan Chen, Pauling Chu, Chia-Chao Wu, Lan Lo, Liang-Kuang Diang, Kuo-Cheng Lu

**Affiliations:** ^1^Department of Medicine, Cardinal Tien Hospital, School of Medicine, Fu-Jen Catholic University, No. 510, Zhongzheng Road, Xinzhuang District, New Taipei City 24205, Taiwan; ^2^Department of Medicine, Show-Chwan Memorial Hospital and Department of Nursing, Meiho University, 542, Sec. 1 Chung-shan Road, Changhua 50008, Taiwan; ^3^Division of Nephrology, Mackay Memorial Hospital and Department of Pharmacology, Taipei Medical University, No. 252, Wu-Hsing Street, Taipei City 11031, Taiwan; ^4^Division of Nephrology, Department of Medicine, Tri-Service General Hospital, National Defense Medical Center, No. 325, Sec. 2, Chenggong Road, Neihu District, Taipei City 11490, Taiwan

## Abstract

Patients on long-term dialysis may develop secondary hyperparathyroidism (SHPT) with increased serum concentrations of bone resorption markers such as the cross-linked N-telopeptide of type I collagen (NTX) and type-5b tartrate-resistant acid phosphatase (TRAP). When SHPT proves refractory to treatment, parathyroidectomy (PTX) may be needed. Renal patients on maintenance HD who received PTX for refractory SHPT (*n* = 23) or who did not develop refractory SHPT (control subjects; *n* = 25) were followed prospectively for 4 weeks. Serum intact parathyroid hormone (iPTH), NTX, TRAP, and bone alkaline phosphatase (BAP) concentrations were measured serially and correlation analyses were performed. iPTH values decreased rapidly and dramatically. BAP values increased progressively with peak increases observed at 2 weeks after surgery. NTX and TRAP values decreased concurrently and progressively through 4 weeks following PTX. A significant correlation between TRAP and NTX values was observed before PTX but not at 4 weeks after PTX. Additionally, the fractional changes in serum TRAP were larger than those in serum NTX at all times examined after PTX. Serum iPTH, TRAP, and NTX values declined rapidly following PTX for SHPT. Serum TRAP values declined to greater degrees than serum NTX values throughout the 4-week period following PTX.

## 1. Introduction

Secondary hyperparathyroidism (SHPT), a common complication of chronic renal disease and of end-stage renal disease under dialysis, results from vitamin D deficiency, impaired mineral metabolism, and decreased serum erythropoietin values [1–3]. Serum parathyroid hormone (PTH) concentrations are elevated such that bone demineralization and bone turnover are enhanced, a condition characterized by the predominance of bone resorption over bone formation [4, 5]. Patients with SHPT are therefore typically treated with vitamin D analogues or calcimimetics. When SHPT is resistant to such treatments, parathyroidectomy (PTX) is indicated; substantial increases in bone mineral density are observed following this surgery [6, 7].

Bone remodeling normally begins with the creation of resorption pits by osteoclasts, followed by osteoclast apoptosis, osteoblast formation, and mineralization [8, 9]. However, this form of remodeling is not observed in patients who have undergone PTX. The pathophysiology of the “hungry bone syndrome” observed after PTX is similar to the bone marrow ablation model used to investigate osteogenic capacity [10, 11]. Accordingly, PTX induces an initial rapid and prominent activation of bone synthetic activity, followed by a suppression of osteoclast activity which persists for at least one year [12].

Several enzymes and type I collagen fragments released during bone formation or resorption have recently been proposed as markers of bone remodeling. Type-5b tartrate-resistant acid phosphatase (TRAP) is an osteoclast-specific enzyme released during the process of bone resorption, and serum concentrations of the enzyme have been found to correlate directly with the number of osteoclasts in bone biopsies. TRAP is therefore proposed to serve as a marker of bone resorption in renal disease [13]. The cross-linked N-telopeptide of type I collagen (NTX), another marker of osteoclastic bone resorption, is measurable in urine or serum [14]. Although renal disease precludes urinary measurements of NTX for hemodialysis (HD) patients, measurement of serum NTX should serve as a valid marker of the bone resorption state for such patients [15].

Persistent suppression of osteoclast function is well-established to follow PTX. However, the relative dispositions of TRAP and NTX and their relationships to other markers of bone resorption have not been established following the surgery. The following study was undertaken to determine serum concentrations of iPTH, NTX, and TRAP at various times following PTX. The main objective was to ascertain whether the post-surgical concentrations of TRAP and NTX are interrelated.

## 2. Methods

### 2.1. Patients

 A total of 48 patients receiving long-term hemodialysis therapy were enrolled in this study, which included 23 subjects with refractory SHPT and 25 subjects who did not develop refractory SHPT (control subjects). Informed consent was secured from all subjects. Patients deemed eligible were between 32 and 72 years of age. Control subjects had serum iPTH values <300 pg/mL; gender and age distributions for the control group were similar to those of the study group. Patients with refractory SHPT were known to have end-stage renal disease of at least 3 months duration and were receiving maintenance HD three times weekly. None of the patients in this study exhibited signs of malnutrition and all had serum aluminum values below 1.0 *μ*mol/L. The study was approved by the Institutional Review Board of the Cardinal Tien and Tri-Service General Hospitals (Taipei, Taiwan).

PTX was considered necessary when (a) optimal medical and dietary treatments for hyperparathyroidism were unsuccessful, (b) high serum iPTH values persisted, and (c) drug-resistant hyperphosphatemia, hypercalcemia, severe osteopathy, vascular calcification, and calciphylaxis were observed. Exclusion criteria included the presence of adynamic bone disease, recent infection, chronic obstructive pulmonary disease, malignant disease, chronic alcoholism, gastrointestinal disease, coronary artery disease, or use of mineralocorticoids, immunosuppressants, or anabolic agents [16, 17]. Patients who had received a renal transplant were also excluded.

The known causes of renal failure were diabetic nephropathy (*n* = 6), hypertensive nephrosclerosis (*n* = 2), chronic glomerulonephritis (*n* = 8), polycystic kidney disease (*n* = 1), and analgesic nephropathy (*n* = 1). Five subjects had renal failure of unknown cause. No subject had received an aluminum-containing phosphate binder for at least one year prior to surgery.

### 2.2. Parathyroidectomy (PTX)

After performance of total PTX, resected parathyroid tissue was divided into pieces of approximately 1 mm in diameter, and a single piece (100 mg of tissue) was auto-transplanted into the subcutaneous fat of one forearm. Following PTX and transplantation of parathyroid tissue, serum iPTH values were <100 pg/mL and serum calcium values were maintained at approximately 8 to 9 mg/dL. If iPTH subsequently rose to values >100 pg/mL, serum calcium was maintained at approximately 9 to 10 mg/dL by administration of low-dose active vitamin D and calcium salts to prevent recurrent HPT. When iPTH values fell below 70 pg/mL, the vitamin D treatment was interrupted to avoid adynamic bone disease [18].

No supplementation with vitamin D was provided to any patient for at least one month preceding surgery. Postoperatively, all subjects received oral calcitriol (1,25(OH)_2_ vitamin D_3_, 2.0 *μ*g/d) [19]. Otherwise, intravenous calcium gluconate was administered postoperatively as needed. Some patients received oral calcium supplements at established daily doses. No cases of permanent hypoparathyroidism were observed.

### 2.3. Biochemical Parameters and Serum Bone Resorption Markers

 To obtain baseline values, fasting (10 h) blood samples were collected between 8 : 00 and 9 : 00 AM preoperatively on the day of PTX (D0). Post-PTX blood samples were collected at 24 and 72 h (D2 and D4, resp.) and at 1, 2, and 4 weeks (W1, W2 and W4, resp.) after the surgery. To harvest serum, blood samples were subjected to centrifugation for 30 min. Serum was obtained within 1 h and stored at −30°C until use for measurements of iPTH, calcium, phosphate, and other bone metabolism parameters.

Serum iPTH concentrations were measured using a two-site immunoradiometric assay (Nichols Institute diagnostics, San Juan Capistrano, CA, USA) which detects the biologically intact 84-amino-acid chain of PTH. Total calcium (TCa), serum phosphate (Pi), alkaline phosphatase, and albumin were determined with an AU5000 automated chemistry analyzer (Olympus, Tokyo, Japan). Serum aluminum was measured by atomic absorption spectrometry in a graphite oven. Serum NTX concentrations were measured using an ELIZA kit (OSTEOMARK; Mochida pharmaceutical Co., Tokyo, Japan); this marker is measured spectrophotometrically and its concentration is determined from a standard calibration curve. Normal NTX values ranged between 6.2 and 19.0 nM bone collagen equivalents/L (BCE/L) for premenopausal women and between 5.4 and 24.2 nM BCE/L for men. Serum TRAP activity was assayed in multi-well plates in the presence of monoclonal antibody (14G6) against the enzyme. Substrate (7.6 mM p-nitrophenyl phosphate in 0.1 M sodium acetate/0.05 M sodium tartrate, pH 6.1) was then added, and samples were incubated for 60 min at 37°C. After termination of the reaction by addition of 3 N NaOH, absorbance at 410 nm was determined using a BioRad 550 microplate reader. The intra-assay variation was 3.7%. To minimize interassay variation, all samples were assayed for TRAP on the same day. BAP concentrations were measured by use of the Osteolinks-Bone ALP high-sensitivity diagnostic ELISA (Quidel, Inc., CA, USA). Normal BAP values ranged between 11.6 and 29.6 U/L for women aged 25–44 y, between 14.2 and 92.7 U/L for women aged >45 y, and between 15 and 40.3 U/L for men aged >25 y.

### 2.4. Statistical Analyses

Continuous variables are expressed as the means ± SD, and categorical values are expressed in percentages. The Shapiro-Wilk test was employed to assess the normality of sample distribution. Differences between pre (D0)- and post (W4)-PTX parameters were evaluated with the paired Student's *t*-test. Differences between groups were evaluated using the analysis of covariance (ANCOVA) test after adjusting for age and sex. Univariate simple regression analysis was used to ascertain whether the relationship between parameters was linear. Spearman's correlation coefficients were calculated assuming nonnormal distribution. A *P* value of <0.05 was considered statistically significant. All statistical tests were performed with the statistical package for social sciences (SPSS, version 17.0) for Windows (SPSS Inc, Chicago, IL, USA).

## 3. Results

### 3.1. Characteristics of the Study Subjects

General characteristics, clinical variables, and biochemical parameters of the control and study groups are shown in Table 1. Compared to the control group, patients with SHPT had higher baseline (pre-PTX, D0) values for iPTH, NTX, TRAP, BAP, TCa, and Pi. At 4 weeks following PTX for subjects with SHPT (W4), serum iPTH, NTX, TRAP, and Pi values decreased significantly. Table 1 also presents the fractional changes (FC, %) in serum bone marker concentrations between D0 and W4. Large negative percentages for iPTH, NTX, and TRAP were observed for the PTX group but not for the control group.

### 3.2. Serum Biochemical Parameter Changes before and after Parathyroidectomy

**Figure 1:**
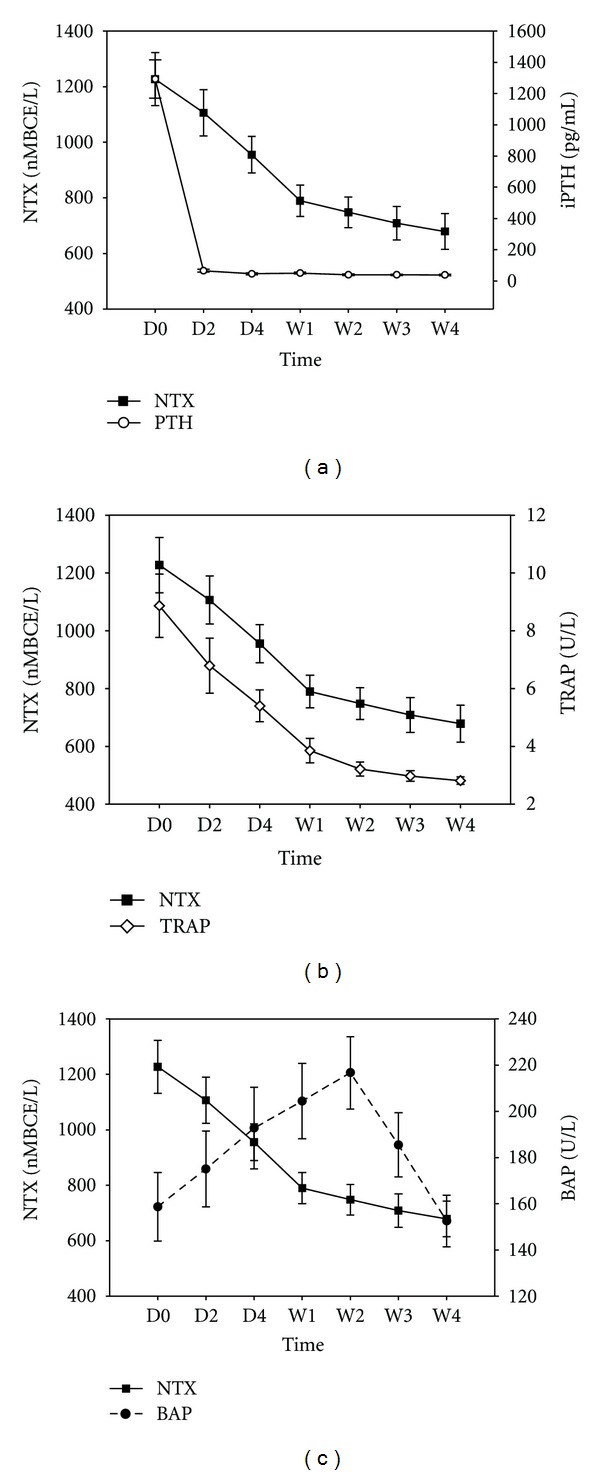
Comparison of serum values for the cross-linked N-telopeptide of type I collagen (NTX) with those for other bone markers before and at various times after parathyroidectomy (PTX) for secondary hyperparathyroidism. (a) NTX and intact parathyroid hormone (iPTH); (b) NTX and tartrate-resistant acid phosphatase 5b (TRAP); (c) NTX and bone alkaline phosphatase (BAP). D0: before PTX; D2, D4, W1, W2, and W4: 1 day, 3 days, 1 week, 2 weeks, and 4 weeks after PTX, respectively. Findings are presented as means ± SEM.

Figure 1 presents serum values for NTX, iPTH, TRAP, and BAP for the study group at D0, D2, D4, W1, W2, W3, and W4. iPTH declined sharply after PTX, with minimum values observed at D2 (D0: 1293.3 ± 591.8 versus D2: 65.2 ± 44.5 pg/mL; *P* < 0.001; panel (a)). NTX values declined rapidly during the first week post-PTX but more slowly during subsequent weeks (D0: 1227.3 ± 458.8 versus W1: 789.5 ± 269.9 nM BCE/L; *P* < 0.001; panels (a), (b), and (c)). TRAP values also declined rapidly during the first week post-PTX but more slowly during subsequent weeks (D0: 8.87 ± 5.27 versus W4: 2.81 ± 0.61 U/L; *P* < 0.001; panel (b)). The disposition of BAP, however, differed considerably from that of iPTH and TRAP. BAP values increased from D0 through W2 (D0: 158.6 ± 71.2 versus W2: 216.7 ± 74.9 U/L; *P* < 0.001; panel (c)). Thereafter, BAP values decreased reaching baseline values at W4 (152.6 ± 53.6 U/L, *P* < 0.001 compared with W2; panel (c)).

### 3.3. Correlation Analyses

To examine potential relationships between NTX and other parameters, measurements obtained at various times were pooled and statistical analysis was performed (Table 2). NTX values correlated significantly with those for iPTH, TRAP, BAP, TCa, and Pi (*r* = 0.450, *P* < 0.001; *r* = 0.690, *P* < 0.001; *r* = 0.448, *P* < 0.001; *r* = −0.161, *P* = 0.042; and *r* = 0.197, *P* = 0.012, resp.).

We calculated univariate Spearman's correlation coefficients to determine the relationships between serum NTX and iPTH, BAP, and TRAP levels at different times after PTX (Table 3).

**Figure 2:**
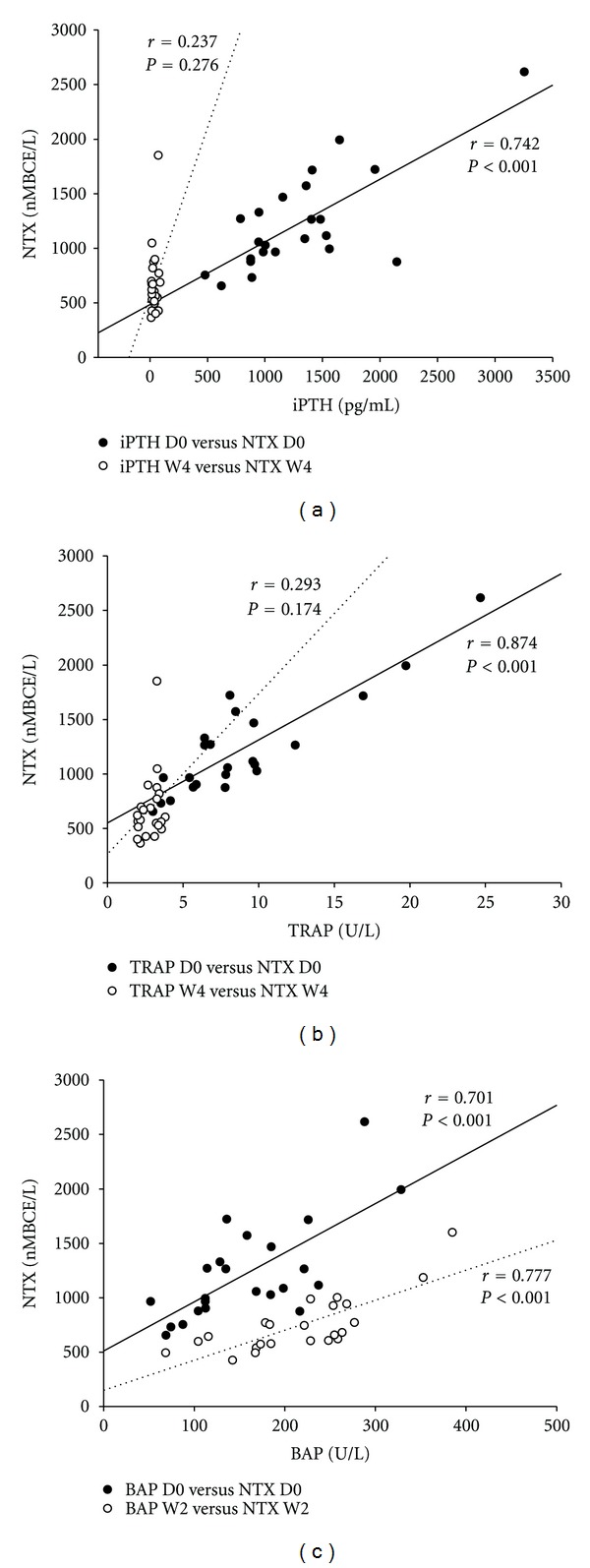
Correlations between serum values for the cross-linked N-telopeptide of type I collagen (NTX) and those for other bone biomarkers before and at 2 or 4 weeks following parathyroidectomy for secondary hyperparathyroidism. (a), Correlation with intact parathyroid hormone (iPTH); (b), correlation with tartrate-resistant acid phosphatase 5b (TRAP); (c), correlation with bone-specific alkaline phosphatase (BAP). D0, before surgery; W2, 2 weeks post-surgery; W4, 4 weeks post-surgery. Spearman's correlation coefficients were calculated assuming a nonnormal distribution.

Figure 2 presents correlations between serum NTX values and those for other bone biomarkers before (D0) and at 4 weeks after PTX (W4). Before PTX, iPTH concentrations were significantly correlated with NTX concentrations (*r* = 0.742, *P* < 0.001; panel (a)). This finding is consistent with the presence of high iPTH values due to increased osteoclast activity as reflected by elevated NTX values. However, this correlation was lost at 4 weeks after PTX (*P* = 0.276). TRAP and NTX values (panel (b)) were also found to correlate significantly before (*r* = 0.874, *P* < 0.001), but not 4 weeks after (*r* = 0.293, *P* = 0.174), PTX. Correlations between NTX and BAP are presented in panel (c). Since BAP concentrations were observed to peak at W2, this time was chosen for analysis. BAP and NTX were significantly correlated both before and 2 weeks after PTX (*r* = 0.701, *P* < 0.001 versus *r* = 0.777, *P* < 0.001, resp.).

**Figure 3:**
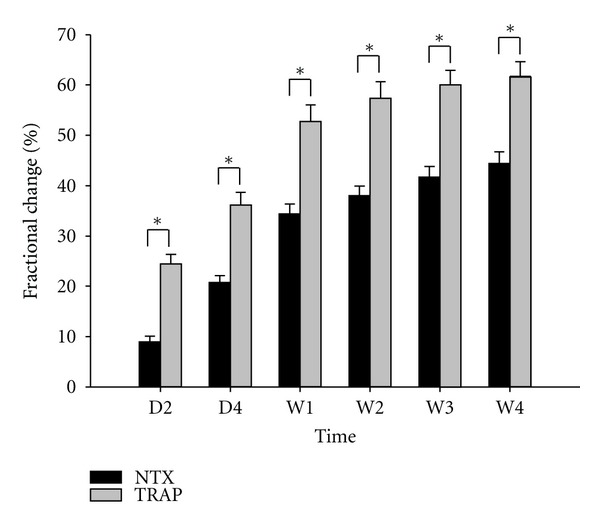
Magnitude of changes in values for serum cross-linked N-telopeptide of type I collagen (NTX) and tartrate-resistant acid phosphatase 5b (TRAP) expressed as mean percent change compared to baseline at 1 day (D2), 3 days (D4), 1 week (W1), 2 weeks (W2), 3 weeks (W3), and 4 weeks (W4) after parathyroidectomy for secondary hyperparathyroidism. *P < 0.001 by the Mann-Whitney Rank Sum Test.

The fractional changes (FC, %) in serum NTX and TRAP concentrations were calculated as a function of time following PTX (Figure 3). FC values for NTX were significantly lower than those for TRAP at all times examined. FC values for NTX versus TRAP were: D2 (−8.94 ± 5.58% versus −24.60 ± 9.06%, *P* < 0.001), D4 (−20.74 ± 6.84% versus −36.30 ± 11.93%, *P* < 0.001), at W1 (−34.42 ± 9.22% versus −52.89 ± 16.00%, *P* < 0.001), W2 (−38.04 ± 9.26% versus −57.55 ± 15.78%, *P* < 0.001), W3(−41.68 ± 10.34% versus −60.21 ± 14.45% *P* < 0.001), and W4 (−44.43 ± 11.02% versus −61.76 ± 13.84%, *P* < 0.001).

## 4. Discussion

The present study is the first to examine a variety of serum markers of bone resorption with respect to their disposition and interrelationships following PTX for SHPT. iPTH concentrations were found to decrease profoundly within 24 h following PTX. Serum NTX and TRAP values declined progressively over the 4-week period following the surgery. By contrast, BAP concentrations rose postoperatively within 24 h and peaked at 2 weeks post-PTX. These findings agree with those of previous studies [20] in which decreases in NTX and TRAP and increases in BAP were observed after PTX for SHPT. Significant correlations of NTX with iPTH, TRAP, BAP, TCa, and Pi were observed in the present study. However, NTX correlated significantly with iPTH and TRAP before (D0) but not at 4 weeks (W4) after surgery. Additionally, the mean percent decrease in serum NTX values was significantly smaller than that in serum TRAP values at all times following PTX. The finding that TRAP and NTX values, both of which reflect osteoclastic activity, did not correlate directly following PTX was unexpected.

 NTX, which is formed during the process of bone resorption by osteoclasts, is proposed to serve as a valid marker of the process [21]. In patients without renal disease, NTX values fluctuate greatly in response to changes in bone resorption patterns and may vary as much as 50% on a daily basis [22]. Although impaired urine production precludes urinary measurement of NTX in HD patients, serum NTX provides an excellent marker of the bone resorption state in these patients [15, 23, 24]. Accordingly, HD patients have high serum NTX concentrations and exhibit rapid rates of bone loss, and NTX values have been found to predict accurately the rate of bone loss over a 2-year follow-up period for these patients [15]. In postmenopausal women, effective bisphosphonate treatment results in a quantifiable reduction in urinary excretion of NTX and improvement in bone mass [25, 26]. In addition, serum NTX has been found useful as a monitor of the anti-resorptive efficacies of pharmacologic treatments for tumor bony metastasis [27, 28]. Findings of the present study agree well with these observations. A significant and progressive decrease in serum NTX values was observed after PTX, indicative of a significant attenuation of bone collagen breakdown. Furthermore, after PTX for primary hyperparathyroidism, a condition in which increased bone resorption is related to excessive secretion of PTH [29]. Serum PTH and NTX concentrations decline rapidly and in parallel; declines in these markers are similar to those observed in the present study after PTX for SHPT.

 Serum TRAP concentration is not influenced by renal function and exhibits less biological variation compared with urinary and serum concentrations of NTX and C-telopeptides of type-I collagen [30]. Increased osteoclastic activity during the process of bone resorption is associated with increased synthesis and secretion of TRAP [31, 32]. TRAP activity is increased in patients with chronic renal failure and in uremic patients with SHPT [33]. Decreases in serum TRAP reflect decreases in osteoclast activity, and serum TRAP is independently correlated with decreased bone mineral density [34]. Furthermore, pre-PTX serum TRAP values have been found to be predictive of bone mineral densities for the femoral neck and lumbar spine at 1-year following PTX [35]. In the present study, a significant correlation between iPTH and TRAP was observed before and after PTX, highlighting the role of PTH in bone resorption in patients with SHPT. The observed decline in serum TRAP activity after PTX therapy is therefore proposed to be attributable to the lower serum iPTH values in these patients.

In maintenance HD or pre-dialysis patients, an elevation in serum BAP is related to increased cardiovascular mortality and vascular calcification [36, 37]. In the present study, an elevation in serum BAP was observed with peak values obtained at 2 weeks post-surgery. This observation may be related to development during the early post-PTX period of the “hungry bone syndrome” associated with increased calcium deposition in bone [38]. The observed increase in serum BAP concentration at these early times may also promote mineralization by extraction of inhibitory pyrophosphate [39, 40].

A significant correlation between BAP and NTX concentrations was observed before and 2 weeks after PTX. This observation is consistent with coupling of osteoblastic bone formation and osteoclastic bone resorption regardless of PTX. By contrast, TRAP and NTX concentrations did not correlate significantly at 4 weeks post-surgery, and the fractional changes in TRAP concentrations did not correlate with those for NTX at any time post-PTX. These findings favor the hypothesis that although osteoclast number is decreased after PTX, those osteoclasts that remain are still capable of degrading collagen. However, renal retention of NTX cannot be excluded. Other limitations of the current study include the small sample size, follow-up is limited to 4 weeks and also the lack of bone histomorphometric data.

 In conclusion, serum iPTH, TRAP, and NTX concentrations decline rapidly following PTX for SHPT. The decline in these markers is attributable to suppression of bone resorption. Fractional decreases in serum TRAP are larger than those in serum NTX during the post-PTX period. Whether the latter observation indicates that bone collagen digestive ability declines more slowly than TRAP activity or reflects the renal retention of NTX warrants further study.

## Figures and Tables

**Table 1:** Clinical variables and biochemical parameters for the control group and for the parathyroidectomy (PTX) group before and 4 weeks after the surgery.

	PTX (*n* = 23)		Control (*n* = 25)	
Age, y	49.3 ± 10.8		49.6 ± 11.4	
Gender (M/F)	11/12		12/13	
HD duration	70.1 ± 21.9 months		68.7 ± 19.3 months	
1,25(OH)_2_ vitamin D_3_	2.0 *μ*g/day		(—)	

	D0	W4	D0	W4

iPTH, pg/mL	1293.3 ± 591.9^b^	38.2 ± 23.6^d^	189.4 ± 75.2^f^	192.4 ± 87.4^f^
NTX, (nM BCE/L)	1227.3 ± 458.8^a^	679.1 ± 307.2^d^	784.1 ± 325.6	769.4 ± 319.6^e^
TRAP, U/L	8.87 ± 5.27^b^	2.81 ± 0.61^d^	2.24 ± 0.65^f^	2.19 ± 0.67^f^
BAP, U/L	158.6 ± 71.2^b^	152.6 ± 53.6^d^	26.5 ± 8.4^f^	27.4 ± 9.1^f^
TCa, mg/dL	10.89 ± 0.86^b^	8.52 ± 0.54^c^	9.5 ± 0.6^e^	9.6 ± 0.6^f^
Pi, mg/dL	6.10 ± 0.48^a^	2.95 ± 0.77^d^	4.9 ± 0.5^f^	5.0 ± 0.6^f^
Albumin, g/dL	3.91 ± 0.36	3.98 ± 0.33	3.95 ± 0.37	3.96 ± 0.36
FC, %				
iPTH		−96.92 ± 1.90		1.58 ± 16.22
NTX		−44.43 ± 11.02		3.40 ± 8.33
TRAP		−61.76 ± 13.84		−2.23 ± 3.08
BAP		−0.75 ± 17.04		−1.87 ± 1.84

^a^ *P* < 0.05, ^b^ *P* < 0.01, compared with control group at D0; ^c^ *P* < 0.05, ^d^ *P* < 0.01, compared with PTX group at D0; ^e^ *P* < 0.05, ^f^ *P* < 0.01, compared with PTX group at W4, Abbreviations: BCE, bone collagen equivalents; BAP, bone alkaline phosphatase; D0, Day 0, before performance of PTX; HD, hemodialysis; W4, 4 weeks after PTX; iPTH, intact parathyroid hormone; NTX, cross-linked N-telopeptide of type I collagen; PTX, parathyroidectomy; Pi, inorganic phosphate; TCa, total calcium; TRAP, tartrate-resistant acid phosphatase 5b; FC: fractional change (%) in marker value between D0 and W4.

**Table 2:** Associations between the cross-linked N-telopeptide of type I collagen and other parameters of bone resorption (measurements obtained at various times were pooled).

	*r*	*P*
iPTH	0.450	<0.001
TRAP	0.690	<0.001
BAP	0.448	<0.001
Ca	−0.161	0.042
Pi	0.197	0.012

iPTH, intact parathyroid hormone; TRAP, tartrate-resistant acid phosphatase 5b; BAP, bone alkaline phosphatase; TCa, total calcium; Pi, inorganic phosphate. Spearman's correlation coefficients were calculated assuming a nonnormal distribution.

**Table 3:** The relationships between serum NTX and iPTH, TRAP, and BAP levels at different times after parathyroidectomy (PTX).

	NTX D0	NTX D2	NTX D4	NTX W1	NTX W2	NTX W3	NTX W4
iPTH D0	*r* = 0.742, *P* < 0.001						
iPTH D2		*r* = 0.236, *P* = 0.279					
iPTH D4			*r* = 0.307, *P* = 0.155				
iPTH W1				*r* = 0.370, *P* = 0.082			
iPTH W2					*r* = 0.314, *P* = 0.145		
iPTH W3						*r* = 0.290, *P* = 0.179	
iPTH W4							*r* = 0.237, *P* = 0.276
TRAP D0	*r* = 0.874, *P* < 0.001						
TRAP D2		*r* = 0.887, *P* < 0.001					
TRAP D4			*r* = 0.700, *P* < 0.001				
TRAP W1				*r* = 0.647, *P* = 0.001			
TRAP W2					*r* = 0.451, *P* = 0.031		
TRAP W3						*r* = 0.412, *P* = 0.051	
TRAP W4							*r* = 0.293, *P* = 0.174
BAP D0	*r* = 0.701, *P* < 0.001						
BAP D2		*r* = 0.643, *P* = 0.001					
BAP D4			*r* = 0.704, *P* < 0.001				
BAP W1				*r* = 0.709, *P* < 0.001			
BAP W2					*r* = 0.777, *P* < 0.001		
BAP W3						*r* = 0.791, *P* < 0.001	
BAP W4							*r* = 0.696, *P* < 0.001

Post-PTX blood samples were collected at 24 and 72 h (D2 and D4, resp.) and at 1, 2, and 4 weeks (W1, W2 and W4, resp.) after the surgery.

Spearman's correlation coefficients were calculated assuming a nonnormal distribution.
